# Chemical deposition of Cu_2_O films with ultra-low resistivity: correlation with the defect landscape

**DOI:** 10.1038/s41467-022-32943-4

**Published:** 2022-09-09

**Authors:** Abderrahime Sekkat, Maciej Oskar Liedke, Viet Huong Nguyen, Maik Butterling, Federico Baiutti, Juan de Dios Sirvent Veru, Matthieu Weber, Laetitia Rapenne, Daniel Bellet, Guy Chichignoud, Anne Kaminski-Cachopo, Eric Hirschmann, Andreas Wagner, David Muñoz-Rojas

**Affiliations:** 1 Univ. Grenoble Alpes, CNRS, Grenoble INP, LMGP, F-38000 Grenoble, France; 2 Univ. Grenoble Alpes, Univ. Savoie Mont Blanc, CNRS, Grenoble INP, IMEP-LaHC, 38000 Grenoble, France; 3 Univ. Grenoble Alpes, CNRS, Grenoble INP, SIMAP, 38000 Grenoble, France; 4 Institute of Radiation Physics, Helmholtz-Zentrum Dresden-Rossendorf, Bautzner Landstrasse 400, 01328 Dresden, Germany; 5 Faculty of Materials Science and Engineering, Phenikaa University, Hanoi, 12116 Vietnam; 6 Catalonia Institute for Energy Research (IREC), Jardins de Les Dones de Negre 1, Barcelona, 08930 Spain

**Keywords:** Structural properties, Electronic properties and materials

## Abstract

Cuprous oxide (Cu_2_O) is a promising p-type semiconductor material for many applications. So far, the lowest resistivity values are obtained for films deposited by physical methods and/or at high temperatures (~1000 °C), limiting their mass integration. Here, Cu_2_O thin films with ultra-low resistivity values of 0.4 Ω.cm were deposited at only 260 °C by atmospheric pressure spatial atomic layer deposition, a scalable chemical approach. The carrier concentration (7.10^14^−2.10^18^ cm^−3^), mobility (1–86 cm^2^/V.s), and optical bandgap (2.2–2.48 eV) are easily tuned by adjusting the fraction of oxygen used during deposition. The properties of the films are correlated to the defect landscape, as revealed by a combination of techniques (positron annihilation spectroscopy (PAS), Raman spectroscopy and photoluminescence). Our results reveal the existence of large complex defects and the decrease of the overall defect concentration in the films with increasing oxygen fraction used during deposition.

## Abstract (web summary)

Cu_2_O offers a lot of potential for several optoelectronic applications. Here, the authors present a low temperature, fast and scalable approach to deposit Cu_2_O films with low resistivity, which is correlated to the defect landscape in the material.

## Introduction

Cu_2_O has been extensively studied due to its appealing optical properties, with a direct bandgap in the range of ~1.93–2.17 eV and a high absorption coefficient of ~10^5 ^cm^−1^ for wavelengths between 510 and 635 nm^1–4^. In addition, the non-toxicity, abundance, and moderate production cost have prompted renewed interest in Cu_2_O, based on its high potential for integration into optoelectronic devices^5–8^. So far, the best transport properties and device performances have been obtained with synthesis methods using vacuum and/or high temperatures (around 1000 °C)^9–13^. The deposition of thin, uniform Cu_2_O films using atmospheric-pressure techniques and low temperatures is of great interest to integrate Cu_2_O in mass-produced devices.

The electrical and optical properties of Cu_2_O thin films strongly depend on their morphology, as well as on the concentration and nature of defects. Therefore, understanding the defect landscape in Cu_2_O is essential for successful integration of Cu_2_O into devices. Several works have reported the impact of parameters like grain size, morphology, and film density on the properties of Cu_2_O for many applications^14–18^. Conversely, limited attention has been given in the literature to experimentally comprehend the impact of intrinsic point defects on the transport properties of Cu_2_O thin films^19,20^. In fact, it is widely believed that the main defects formed are copper vacancies in the normal (V_Cu_) and split configuration (V_Cu,split_), due to their low formation energy, without taking into account the role of other possible defects in the structure^21–26^. Hence, comprehending the nature and role of the defects in Cu_2_O thin films using techniques that can directly probe them is necessary to better understand the physical phenomena occurring at the quantum level and allow a good integration in electronic and optoelectronic devices^2,27^.

Atomic layer deposition (ALD) is a technique allowing the low-temperature (i.e., below 300 °C) deposition of thin films with sub-nanometric control over thickness, high uniformity, and conformity for high aspect-ratio features and porous substrates^28,29^. However, ALD is usually performed under vacuum and has a very slow deposition rate. Spatial atomic layer deposition (SALD) is a novel approach to ALD that does not require a vacuum and is much faster than conventional ALD by up to two orders of magnitude. This technique can reach the deposition rates of Chemical Vapor deposition (CVD, ie. 1–10 nm/s)^30,31^. SALD has therefore generated much interest for the fabrication of functional devices^32–37^. Indeed, we have recently reported the deposition of Cu_2_O thin films by atmospheric-pressure SALD (AP-SALD), with high mobility values (92 cm^2^/V.s), but still more resistive than Cu_2_O films deposited by physical methods^38^. In the present study, we demonstrate that the electronic and optical properties of the films can be easily tuned by introducing oxygen during the deposition process, reaching ultra-low resistivity values of 0.4 Ω.cm for films deposited at only 260 °C with an oxygen fraction of 15%. We also show that the optical and transport properties of the films can easily be tuned by adjusting the oxygen fraction. Films with a range of carrier concentrations between 7.10^14^ and 2.10^18^ cm^−3^, and mobility ranging from 1 to 86 cm^2^/V.s were obtained for films deposited from 0 to 25% oxygen fraction, respectively. The optical bandgap was observed to change from 2.48 to 2.2 eV.

To correlate the electronic properties of the films with temperature and oxygen fraction, we employed positron annihilation spectroscopy (PAS) techniques to probe the size and concentration of defects in the different films. To the best of our knowledge, this is the first time positron-based techniques are used to study the defects in Cu_2_O thin films. PAS is non-destructive and uses positron-electron annihilation characteristics (positron lifetime and energy distribution of photons resulting from the annihilation process) to probe the volume and concentration of defects^39^. We show that, contrary to what it is currently assumed, large complex defects are the dominant defects in the films. The concentration of such complex defects is very sensitive to deposition conditions, decreasing when oxygen fraction during deposition is increased. These results are supported by photoluminescence measurements and Raman results, as detailed below.

## Results

### Optimization of Cu_2_O by varying the oxygen fraction

**Fig. 1:**
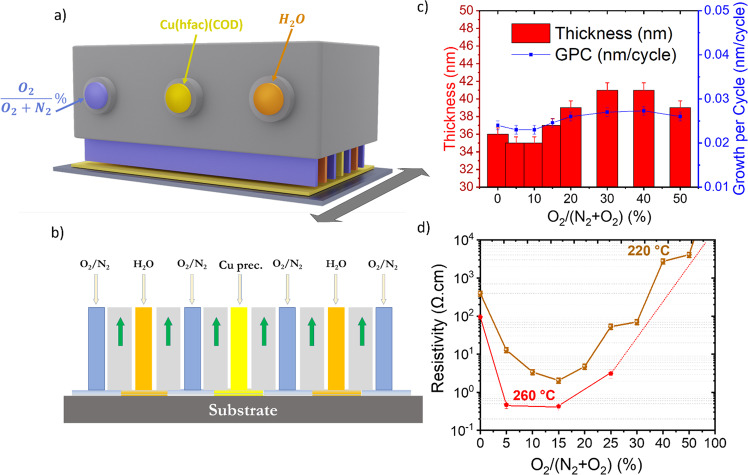
Optimisation of Cu_2_O by varying the oxygen fraction. **a** Schematic of the AP-SALD system used for the deposition of Cu_2_O thin films with the distribution of the flows inside the head (Yellow color corresponds to the (Cu(hfac)(cod)) precursor, the orange color corresponds to the water flow, and the blue color corresponds to the mixture of the nitrogen and oxygen flow). **b** Schematic view of the cross-section of the gap between the head bottom and the substrate. **c** Growth rate and thickness variation of Cu_2_O samples deposited at 220 °C on a glass substrate. **d** Van der Pauw measurements of Cu_2_O samples deposited at 220 °C and 260 °C with different oxygen fractions. Error bars represent min/max values obtained for each point from two different deposition runs.

Figure 1a shows a 3D scheme of the SALD head used to deposit the films in this study. Three incoming gas flows carrying a Cu metalorganic precursor (Cu(hfac)(cod)), water, and N_2_ are distributed along parallel channels on top of the substrate. The latter is placed at close proximity of the head (50–200 μm) to ensure efficient separation of the reactants by the N_2_ flow. The substrate is then scanned back and forth to expose its surface to the reactants, to reproduce typical ALD cycles (Fig. 1b, see Methods, Huerta, C.A.M, et al.^40^ and Nguyen, V.H*,* et al.^41^ for more details). In the present study, Cu_2_O thin films were deposited using different oxygen fractions (0% to 50%). The controlled exposure of the growing film to oxygen was achieved simply by adding O_2_ to the N_2_ flow separating the two reactants (Fig. 1b). Figure 1c shows the thickness and growth rate (growth per cycle, GPC) of Cu_2_O films deposited at 220 °C after 750 oscillations of the substrate under the head (1500 ALD cycles) for different oxygen fractions. The film thickness increases from 35 nm to 41 nm when oxygen fraction increases from 0% to 50%. This corresponds to a GPC higher than for conventional ALD, varying from 0.023 nm/cycle to 0.027 nm/cycle^38^.

Figure 1d presents the corresponding resistivity values of the different films, showing a strong impact of oxygen fraction on conductivity. The resistivity decreases by two orders of magnitude, from 4.10^2^ Ω.cm to 2 Ω.cm, by increasing the oxygen fraction from 0% to 15%. The resistivity then starts to increase with increasing oxygen fraction until it reaches values above 10^3^ Ω.cm for a 50% oxygen fraction, beyond which the films were too resistive to be measured with our Van der Pauw setup. There is thus an optimum oxygen fraction of 15% for which the films present a minimum resistivity, which is one order of magnitude lower than for previous studies on the SALD deposition of Cu_2_O^37,38^. In these previous studies, temperature is another main factor affecting the final transport properties of the films. Films were thus deposited at temperatures ranging from 180 to 260 °C using a 15% oxygen fraction and, as expected, the resistivity of the films decreased with increasing deposition temperature (see supplementary Fig. 1a). Films were then deposited at 260 °C with different oxygen fractions (0, 5, 15, 25, and 50%) to compare the transport properties with the films deposited at 220 °C. As shown in Fig. 1d, the resistivity of these films is lower than for all the corresponding films deposited at 220 °C with the same oxygen fraction (the films, in this case, had a thickness of ~100 ± 5 nm after 3000 ALD cycles, GPC of 0.033 nm/cycle).

**Fig. 2:**
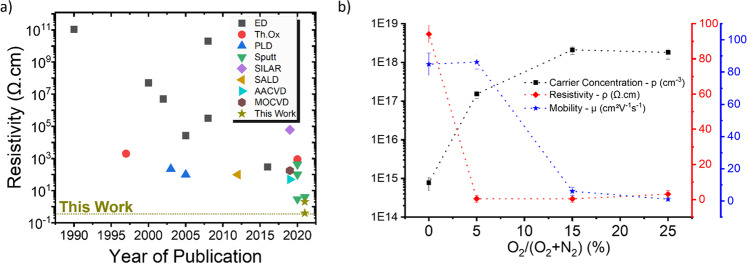
Transport properties. **a** Comparative study of resistivity for different growth techniques (ED^72–78^, Th.Ox^79, 80^, PLD^42, 43^, Sputt^9–11^, Silar^45^, SALD^37^, AACVD^81^, MOCVD^44^ vs. year of publication). **b** Mobility, resistivity, and carrier concentration of Cu_2_O thin films (100 nm) deposited at 260 °C with different O ratios. Error bars represent min/max values obtained for each point from two different deposition runs.

Although the evolution of the resistivity follows a similar trend for both temperatures, the decrease of resistivity upon adding oxygen is more abrupt for the films deposited at 260 °C, decreasing from 94 Ω.cm at 0% to 0.4 Ω.cm at 5% and 15%, and then increasing to 3.11 Ω.cm at 25%, beyond which the sample became too resistive to measure with our setup. Thus, for 260 °C, the minimum resistivity is obtained for 5 and 15% oxygen fractions. Such a low resistivity value is remarkable, especially taking into account that an atmospheric chemical deposition method is used. Indeed, this value represents, to the best of our knowledge, the lowest value obtained in the literature even when comparing with Cu_2_O films deposited by physical methods. Previously, low resistivity films have been deposited using expensive techniques such as pulsed laser deposition (PLD) and sputtering (high-temperature deposition/vacuum processing) or by incorporating extrinsic defects into the films^9–11,42–44^. In our case, very conductive films were obtained using a scalable open-air and low-temperature approach by simply inducing an oxygen fraction into the process. Figure 2a displays the evolution of the resistivity of Cu_2_O thin films over time and for different deposition methods.

### Transport and structural properties

Figure 2b shows the carrier concentration and mobility values for the films deposited at 260 °C with different oxygen fractions (obtained by Hall Effect measurements on samples having a surface area of 1 cm² and gold metal evaporated on the edges). The mobility is quite stable for samples deposited between 0% and 5% oxygen fraction, around 85 cm².V^−1^.s^−1^. It then sharply decreased to values ~6 cm².V^−1^.s^−1^ at 15% and stays relatively the same at 25% oxygen fraction, while the hole density shows an inversed trend, increasing suddenly from 7.10^14^ cm^−3^ at 0% to 1.10^17^ cm^−3^ at 5%. Further, the hole density then stabilizes at 15% and 25%, around 10^18 ^cm^−3^. This shows that the introduction of an oxygen fraction during the deposition process not only improves the conductivity of the films, but also allows tuning of the transport properties of the films. Samples deposited with 5% and 15% oxygen fraction, despite showing the same resistivity value, have very different mobility and carrier concentration values. As is the case for deposition temperature (Supplementary Fig. 1b–d), the optical properties of the films are also affected by the oxygen fraction used and, for example, the bandgap decreases from 2.48 to 2.2 eV when increasing the oxygen fraction (Supplementary Fig. 2). This evolution offers some potential for applications requiring different optical-electrical properties.

**Fig. 3:**
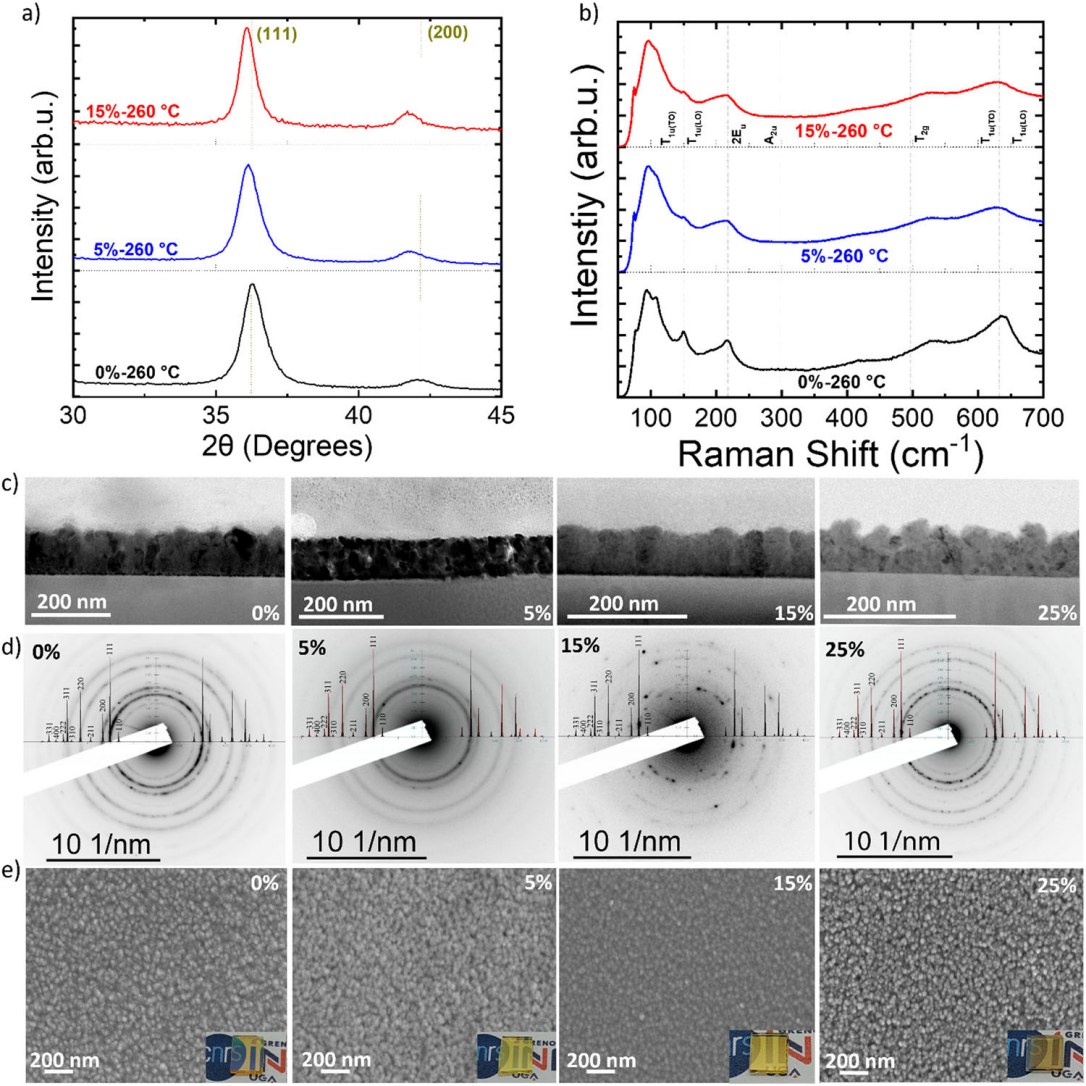
Structural, morphological and optical characterization. **a** GI-XRD, and **b** RAMAN measurements, of samples deposited at 260 °C with 0%, 5% and 15% oxygen fraction. **c** Cross-sectional TEM analysis, **d** Selected area diffraction patterns from plane view, and **e** SEM pictures of Cu_2_O thin films deposited on a borosilicate glass substrate at 260 °C with different oxygen fractions (0%–5%–15%–25%). (Insets show optical images of the corresponding films).

A complete characterization of the films was performed to try to understand the change observed in the transport properties. The results for the films deposited at 260 °C are shown in Fig. 3, while the data for the films deposited at 220 °C are shown in the Supplementary Information. Figure 3a presents the XRD patterns of samples deposited at 260 °C with 0% and 15% oxygen fraction (Supplementary Fig. 3a for the films deposited at 220 °C). The patterns show two peaks corresponding to the (111) and (200) crystalline planes of Cu_2_O, for both temperatures. When increasing the oxygen fraction, two phenomena are observed: the peaks show a shift to lower angles (indicating a change in the cell parameters) and the texture of the films evolves. The shifting of the peaks is reverted for oxygen fractions above 15% (Supplementary Fig. 3a). Thus, adding oxygen to the process induces an initial cell expansion, which is reverted upon increasing the oxygen fraction. Raman spectroscopy was also used to characterize our films. In addition to providing information on the different Cu_2_O phases present in the films, we recently demonstrated that Raman spectroscopy is a powerful tool to probe the concentration of defects in Cu_2_O^38^. Indeed, most peaks in the Raman spectrum of Cu_2_O are due to the presence of intrinsic defects. As such, their intensity can be used to assess the amount of defects in the films^38^. The Raman spectra of Cu_2_O films deposited at 260 °C with 0% and 15% oxygen fractions are presented in Fig. 3b (Supplementary Fig. 3b for 220 °C). It can be clearly observed that for both temperatures the addition of oxygen induces a decrease of the T_1u_ (transversal and longitudinal) and the second-order 2E_u_ modes related to copper vacancy defects in the normal and split configuration (Supplementary Fig. 3c, d for more details)^22^. From our previous study, such a decrease implies that the concentration of copper vacancies, and in particular V_Cu,split_, decreases when adding oxygen^38^. Therefore, the mobility should increase with oxygen fraction, but this is only the case up to an oxygen fraction of 5%. Conversely, a further increase in oxygen fraction results in a sharp decrease of the mobility that cannot be explained with the Raman data obtained. More characterizations were performed to further understand this phenomenon.

Other factors could explain the evolution of the transport properties, such as the morphology of the films, which can be affected by both the deposition temperature^45,46^ and the oxygen fraction. Figure 3c shows TEM cross-section images of the samples with different oxygen fractions. In all cases the films were uniform and dense. Selective area diffraction patterns (SAED, Fig. 3d) obtained in plane view from particles scratched off from the films do not show other phases, namely CuO or Cu, in agreement with the Raman data. SAED images indicate a higher crystallinity of the sample deposited with 15% oxygen fraction. This is confirmed by the crystallite size values obtained from XRD data (Supplementary Table 1). The morphology of the films also evolves with oxygen fraction. At 0% the films display a columnar growth, while at 5% oxygen fraction the films are made of smaller particles and present a smoother surface. The morphology evolution continues for higher oxygen fractions, and the films deposited with 15% oxygen fraction present again columnar growth, while the films deposited with 25% oxygen fraction seem to be composed of tiny particles. The uniformity of the films is shown through SEM pictures in Fig. 3e. Pictures of the different samples are shown in the insets of Fig. 3e. As presented, the film deposited with 25% oxygen fraction displays a darker color, in agreement with the optical characterizations presented in Supplementary Fig. 2.

XPS measurements were also performed for the film deposited at 260 °C with a 15% oxygen fraction (the one showing the lowest resistivity), and showed that no Cu^2+^ is present, as shown in Supplementary Fig. 4. The survey spectrum proves that the film does not contain carbon or other impurities. Another important feature to consider is the mass density of the films at different oxygen fractions. Supplementary Fig. 5a shows the X-ray Reflectometry (XRR) patterns and the deduced density of the different Cu_2_O films. The density at 0% and 5% is 5.8 ± 0.2 g/cm^3^, similar to the single-crystal Cu_2_O density. A decrease to 5.45 ± 0.2 g/cm^3^ is observed for the film deposited with a 15% oxygen fraction. The film densities for all the samples are quite high. Interestingly, the density increases again for a 25% oxygen fraction to a value of 5.8 ± 0.2 g/cm^3^, again very close to the value for Cu_2_O single crystals (6 g/cm^3^). The introduction of the oxygen fraction within the cuprous oxide seems to alter considerably not only the transport properties of the films but also their physicochemical structure. AFM measurements for films deposited with different oxygen fractions (Supplementary Fig. 6) confirm the evolution in roughness observed in cross-sectional SEM images, as the RMS values are 3.9 nm at 0%, decrease to 3.1 nm at 5%, and increase to 5.4 nm at 15%. However, it is still not possible to correlate the observed morphological features of the films (particle size, film texture, and density) with the evolution of the transport properties. Indeed, it has already been shown that the transport properties of Cu_2_O are affected mainly by the nature and concentration of defects present in the grains, rather than the grain boundaries, conversely to what is observed for instance for ZnO and Al:ZnO thin films^32,47^. We believe this is still the case here, despite the different transport regime we obtained with ultra-high conductivity values. Indeed, as shown in our previous work, Cu_2_O samples deposited in the same conditions and having similar grain boundaries and morphology but obtained from different precursors display different transport properties due to the different defect landscape, as deduced from the Raman vibrational modes related to defects^38^.

### Study of the defect landscape evolution by PAS

While there are several cases in the literature dealing with the theoretical study of defects in Cu_2_O^2,22,26,48–50^, there is a lack of experimental evidence directly probing the volume and concentration of defects in Cu_2_O thin films. Theoretical studies indicate that Cu_2_O tends to present free holes as a result of shallow point defects, i.e., V_Cu_ and V_Cu,split_ (copper vacancies in the so-called split configuration)^25^. This is also due to the presence of anti-bonding Cu^(I)^(d^10^) orbitals at the valence band maximum, which results in a lower electron density between the nuclei^26^. Additionally, the p-type nature of the metal oxide is intrinsically stable due to the lack of effective donors inside the bandgap, thus minimizing the compensation effect^26^. Although we have shown that Raman spectroscopy is a powerful tool to assess the relative concentration of defects between different films, the nature of such defects has been studied by DFT^22^ and their concentration cannot be obtained from the spectra^38^.

PAS techniques were used to get direct insight into the size and concentration of defects in the Cu_2_O films deposited in this work. In particular, positron annihilation lifetime spectroscopy (PALS) and Doppler broadening variable energy positron annihilation spectroscopy (DB-VEPAS) were employed (see methods and supporting information, Supplementary Section 2.2). In these techniques, the samples are exposed to positrons (the antiparticle of an electron, having the same mass and positive charge) emitted from a radioactive Na^22^ isotope or via pair production^39,51^. The positrons recombine (annihilate) with electrons in the films, producing at least two gamma photons with a total energy of 1022 keV. In PALS, the time between positron generation and detection of one of the gamma quanta gives direct information on the size (volume) of the defects (Supplementary Section 2.2.3)^51^. On the other hand, in DB-VEPAS the shape of the gamma radiation peak at 511 keV, generated by the annihilation, is affected by the momentum of the electrons involved in the annihilation. Two contributions can be differentiated: from valence electrons, hence open volume defects (S-parameter), or from core electrons (W parameter). In both techniques, the kinetic energy of the positrons can be varied, which results in a different implantation depth that increases with positron energy, thus allowing to do depth-profile analysis. In addition, these techniques are non-destructive and particularly sensitive to defects^51^. For instance, only recently the use of positron annihilation techniques has allowed us to demonstrate that Sr and Ti-O vacancy complexes are manifested in Fe:SrTiO_3_^52^ and Ti single vacancies as well as larger Sr vacancy clusters are more abundant in undoped SrTiO_3_^52^. The feasibility of PAS as a sensitive probe of different defect species has been demonstrated in other oxides as well, particularly in ZnO^53^ and Sr_2_FeMoO_6_^54^. To correlate the PALS and DB-VEPAS data with actual defects in the films, DFT calculations were performed (Supplementary Section 2.2). Note that the DFT calculations performed did not take into account the possible relaxation of the structure due to the defects and thus the obtained defects and associated volumes should be considered as approximate. A precise description of the nature of such complex defects requires more complex DFT calculations, which are out of the scope of this work.

**Fig. 4:**
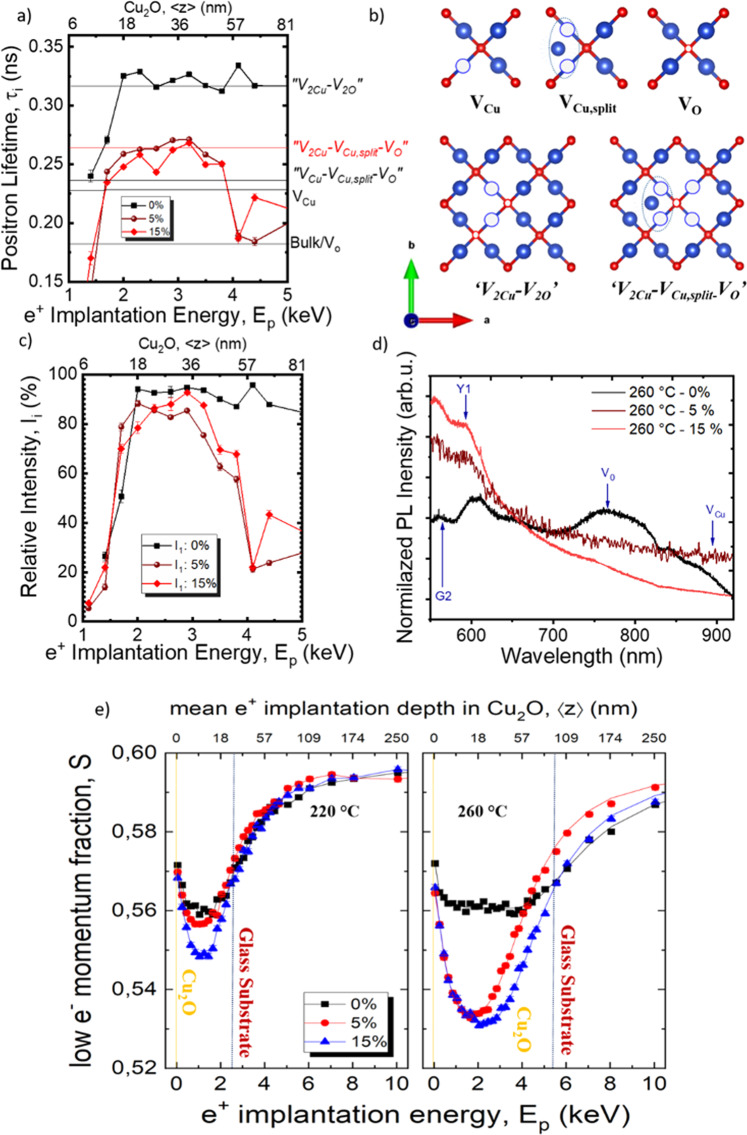
PAS results. **a** Lifetime components and **b** Schematic model of previously reported point defects in Cu_2_O and of much larger complex defects that are expected (after our DFT calculations) to be at the origin of the large positron lifetimes observed in the PALS experiments: copper vacancy in the normal and split configuration (V_Cu_, V_Cu,split_), oxygen vacancy (V_O_), and complex defects “*V*_2Cu_−*V*_2O_”, “*V*_2Cu_−*V*_Cu,split_-*V*_O_” (Blue and red atoms represent the Cu and O atoms, respectively—Vesta), **c** Relative intensities for three different oxygen fractions: 0%, 5%, 15% at 260 °C for 3000 ALD cycles, **d** Normalized photoluminescence measurements displaying exciton, oxygen and copper vacancies, for three different oxygen fractions: 0%, 5%, 15% at 260 °C, and **e** S(Ep) and VEPfit fitted^59^. S(Ep) dependencies for 220 °C (38 nm thick) and 260 °C (100 nm-thick) film series deposited on a glass substrate with different oxygen fractions (0%, 5%, and 15%).

Figure 4a shows the positron lifetime vs. implantation energy (i.e., depth, see top axis) for films deposited with 0, 5, and 15% oxygen ratio. As it can clearly be observed, upon the introduction of oxygen in the deposition process, the positron lifetime decreases, implying that defect size decreases. The shortest positron lifetime component is τ_1_ ≈ 320 ps from samples deposited with a 0% oxygen fraction. Based on the defects expected from theoretical calculations, i.e., V_O_, Cu_2_O crystals should present a positron lifetime of 175 ps, which is not the case for our films. Old studies on single crystals suggested that the lifetimes of about 320 ps would correspond to *V*_Cu_ − *V*_O_ pairs^55^. However, as revealed by our DFT calculations (see Supplementary Section 2.2.4), the volume associated with such a lifetime would correspond to the presence of larger complex defects. To the best of our knowledge, the presence of such large defects has never been proposed for Cu_2_O. The bulk positron lifetime (which would correspond to positron annihilation at interstitial positions) was not detected^56^, suggesting a large initial defect concentration. Indeed, it has been shown that beyond a certain defect concentration positrons always annihilate in a vacancy^39^. Similarly, the lack of positron lifetimes corresponding to smaller defects such as V_Cu_ or V_Cu,split_ does not imply that they do not exist. (Note that a higher positron lifetime component τ_2_ varying from 350 to 500 ps was also obtained. This corresponds to larger vacancy clusters (>5 vacancies within a cluster) and voids that are typically attributed to grain boundaries). Our calculations indicate that the large complex defects responsible for the observed long positron lifetimes could consist of two V_Cu_ and two V_O_, namely, *“V*_*2Cu*_ *−* *V*_*2O*_*”*.

When increasing the oxygen fraction to values of 5% and 15%, a strong reduction of the positron lifetime to τ_1_ = 244–271 ps is observed for both films, being slightly shorter for the films deposited with a 15% oxygen fraction. These values are, however, still larger than what would be expected for *V*_O_ and *V*_Cu_, which again implies the presence of larger complex defects^55^. Based on our DFT calculations, such lifetimes could correspond to a complex defect made of copper vacancies in normal and split configuration *V*_Cu_, *V*_Cu,split_ and oxygen vacancies *V*_O_. Again, this does not imply that there are no individual *V*_O_ or *V*_Cu_ in the film, but that all positrons annihilate in the larger complex defects.

Figure 4b shows a 2D scheme of the known simple defects present in Cu_2_O and the larger complex ones deduced from our PALS data (see Supplementary Fig. 7). Figure 4c presents the relative intensity of respective lifetime components for the different films, as a function of implantation energy. It can clearly be seen that the intensity tends to decrease as the oxygen fraction increases, implying that fewer positrons annihilate with the lifetime τ_1_, and thus that the concentration of defects decreases when increasing the oxygen fraction. Our results therefore suggest a decrease of the overall defect concentration, in agreement with Raman results. It is worth mentioning that above 60 nm, PAS starts to detect the substrate, and the lifetime and its intensity decrease.

Photoluminescence (PL) measurements were performed to gain further insight into the evolution of the defect landscape, as shown in Fig. 4d. The PL spectra of Cu_2_O typically present exciton-related peaks (both Y1 and G2 at 630 and 570 nm respectively) and peaks associated with V_O_ and V_Cu_ at 760 nm and 910 nm, respectively^57^. In our case, films deposited with 0% oxygen fraction show a clear peak corresponding to *V*_O_, and a shoulder at higher wavelength corresponding to V_Cu_, while the excitonic peaks are rather low. Upon increasing the oxygen fraction to 5% and 15%, a clear decrease in the *V*_O_− and *V*_Cu_-related peaks is observed, along with an increase of the intensity of the excitonic peak, thus in agreement with positron and Raman results.

While the PALS technique probes the defect size present in the lattice, giving qualitative information on the relative concentration of defects when comparing different films, the DB-VEPAS technique provides information on the chemical composition at the vacancy-like defects, and allows us to calculate defect densities considering the effect of positrons back-diffusion to the surface. The energy of the emitted photons is measured by means of one or two high-purity Ge detectors (energy resolution of 1.09 ± 0.01 keV at 511 keV). The broadening of the annihilation spectrum is described by the so-called S-parameter, defined as a fraction of the annihilation line in the middle (511 ± 0.93 keV) of the spectrum. The S-parameter represents a fraction of positrons annihilating with low momentum valence electrons and vacancy type defects with their concentration. Plotting the calculated S as a function of positron implantation energy S(E_p_) provides depth-dependent information^58^. Since the S-parameter decreases with the increase in oxygen fraction for both temperatures (Fig. 4e), a reduction in overall defect concentration is expected. The initial drop of S at *E*_p_ < 2 keV indicates back diffusion of positrons to the surface, hence the so-called surface states. Positrons at the surface live much longer compared to bulk, which is reflected in the increased S. After the initial drop, S saturates or exhibits a minimum, which represents the film bulk. The following increase (*E*_p_ > 2 keV for 220 °C and *E*_p_ > 3 keV for 260 °C) indicates an increasingly larger positron fraction annihilating with the substrate. The curve of S-parameter as a function of positron energy S(E_p_) dependencies was fitted with the VEPfit code^59^, and is shown in Table 1. More details on the technique used for this measurement are provided in the Supplementary Section 2.2.1. The fit enables the calculation of an effective S-parameter of the films and compare it with the experimentally measured width of the annihilation line. The fitting enables the analysis of positron diffusion length, L_+_, as shown in Table 1, which is inversely proportional to the concentration of defects (per atom, for more details refer to Supplementary Section 2.2.2). The S(E_p_) was plotted for samples deposited at 220 and 260 °C, with an average thickness of ±38 nm and ±100 nm, respectively.

**Table.1:** Calculated using VEPfit code^59^ S-parameter, effective diffusion length, L_+_, and estimated weighted average overall vacancy defects concentration of Cu_2_O film with a different oxygen fraction at 260 °C

Sample ID	S-parameter	Diffusion length L_+_ (nm)	Estimated defect concentration (C_V_ x 10^−5^)
0%	0.561(1)	3 ± 5	472
5%	0.523 (5)	25 ± 8	6.66
15%	0.516 (4)	31 ± 5	4.28

Samples deposited at these two temperatures with 0% oxygen fraction display the largest S-parameter and the shortest diffusion length. Details on the calculation of the defect concentration are presented in Supplementary Section 2.2.5. These results imply a high defect concentration of the complex defects, as stated previously in the PALS measurement for samples deposited at 260 °C. As the oxygen fraction increases, the S decreases and the L_+_ increases, suggesting a large reduction in defect concentration. Yet, the difference from 0% to 5% is different when depositing the film at 220 or 260 °C. This may indicate that the defects represent a strong decrease only at a higher thermal budget. In summary, all our results, (PALS, DB-VEPAS, Raman, and PL), have demonstrated that the films deposited without any intentional presence of oxygen have a large concentration of defects, which decreases upon controlled addition of oxygen during the deposition. In particular, *V*_O_ are the ones expected to decrease the more (which makes sense given the higher oxygen fraction), as confirmed by PL data.

## Discussion

Looking back at the transport properties of the different films presented in Fig. 2b, we correlate these with the results obtained by PAS, Raman, and PL. For the films deposited with no oxygen, a certain concentration of defects is present (Table 1), which is, in any case, lower than for previous studies in which another precursor had been used, which explains the very good resistivity values already obtained for a 0% oxygen fraction^37,38^. An increase of the oxygen fraction to 5% during the deposition results in a reduction in the concentration of defects, in particular of the large complex defects.

As a result, the corresponding Raman spectra show a clear decrease in intensity (more pronounced for the films deposited at 220 °C, as expected). Interestingly, the mobility of the film is very similar to the one obtained at a 0% oxygen fraction. The reduction of the resistivity is indeed due to an increase in carrier (holes) concentration. Being anionic defects, *V*_O_ act as hole killers, recombining with holes and decreasing the carrier concentration in Cu_2_O thin films^23,26,60^. The increase in carrier concentration observed when oxygen is added during the deposition is thus correlated with a decrease in *V*_O_ concentration in the film, as deduced from our positron study and PL data. Although the mobility is similar for both samples, we expect the concentration of V_Cu,split_ to decrease based on both Raman and positron results. This should result in increased mobility values^38^, but the increase in carrier concentration leads to a higher charge scattering (in particular through the trap-limited conduction caused by the non-spherical Cu 3d orbitals, which causes a strong tailing at the valence band^32,61,62^).

**Fig. 5:**
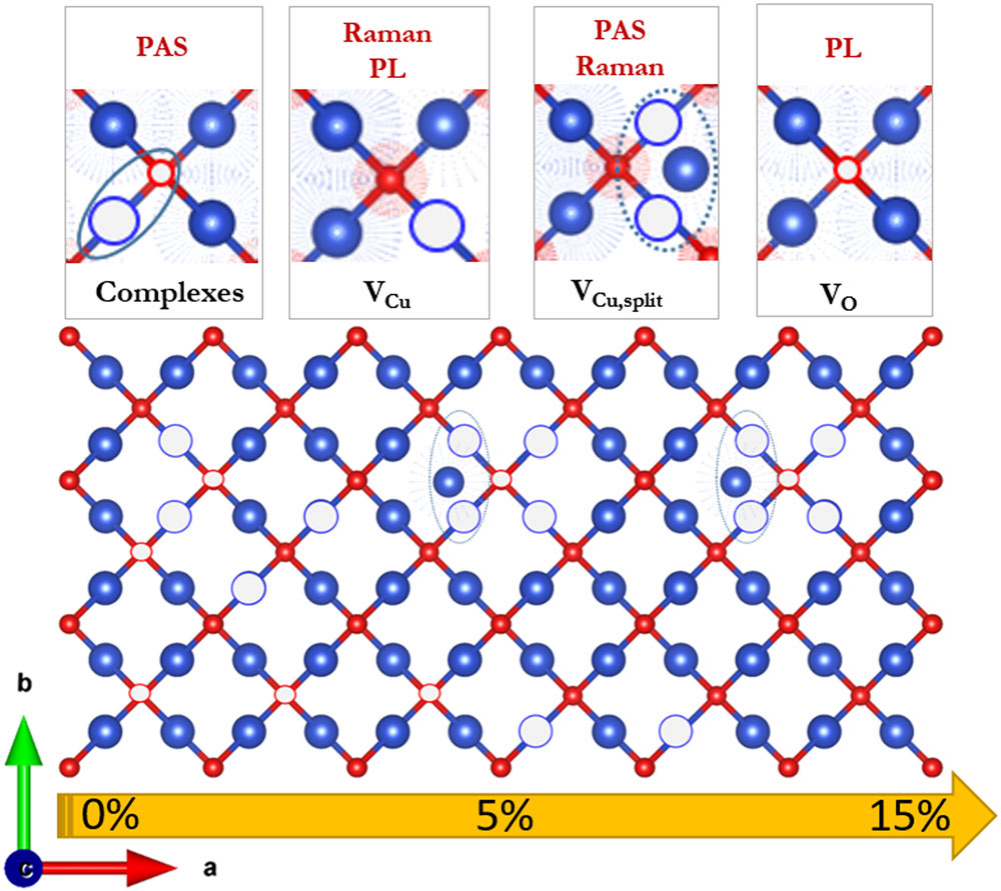
Defect Landscape evolution. Summary of the evolution of defects in the Cu_2_O thin films deposited with different oxygen fractions and the methods used to probe them: complex defects (“*V*_2Cu_-*V*_2O_” and “*V*_2Cu_-*V*_Cu,split_-*V*_O_”), oxygen vacancies (*V*_O_), and copper vacancies in the normal and split configuration (*V*_Cu_, *V*_Cu,split_).

Indeed, given that the concentration of *V*_O_ diminishes to a greater extent with respect to V_Cu,split_, the net charge concentration increases, thus altering the transport properties. Comparing samples with 0% and 5% oxygen fraction, the mobility tends to keep a similar value, while the carrier concentration increases and the resistivity strongly decreases. This could be explained by the integration of the host-derived acceptor level in the film, which significantly changes the resistivity of the film^26^. The role of vacancies on the conductivity of Cu_2_O thin films and single crystals is well documented^23,24,63,64^. For films deposited with a higher oxygen fraction (15% and above), the carrier concentration keeps increasing due to the further reduction in the concentration of *V*_O_. Tabushi. N*.* et al., have demonstrated the opposite effect using a reducing hydrogen agent; the carrier concentration was reduced while the mobility and resistivity gradually increased^65^. In our case such increase has an impact on the mobility obtained, which decreases significantly by an order of magnitude. In any case, the combination of carrier concentration and mobility yields a low resistivity, comparable to the one obtained for films deposited with a 5% oxygen fraction. It also provides an opportunity to tune the transport properties of the films for different applications (i.e., low carrier concentration and high mobility for transistors vs. high carrier concentration to have a short depletion layer in semitransparent junctions). Overall, as the oxygen fraction increases, PL demonstrate a decrease in *V*_O_ defects, while Raman shows a reduction in V_Cu_, in agreement with the measured transport properties. An illustration representing the evolution of the defects during the addition of oxygen is presented in Fig. 5. The combination of all the characterizations implies that those defects decrease upon oxygen induction (as shown in Fig. 5). V_Cu_ concentration becomes higher than *V*_O_ concentration as the oxygen fraction is increased, since these are the main defects seen by the PAS.

In summary, in this work we report the deposition of Cu_2_O thin films with ultra-low resistivity values as low as 0.4 Ω.cm, using a chemical, open-air and scalable approach at temperatures below 260 °C. We show that the introduction of an oxygen fraction during deposition has a strong impact on the properties of the films, including the morphology, crystallinity, bandgap, and transport properties. In particular, the conductivity, carrier concentration, and carrier mobility can be modified by simply adjusting the oxygen fraction used during deposition. We show that as the oxygen fraction increases, the mobility decreases (from 86 to 1 cm^2^/V.s) and the carrier concentration increases (from 10^14^ to 2.1018 cm^−3^). The most conductive films are obtained at 260 °C with oxygen fractions of 5 and 15%. PAS has been used for the first time to probe the nature and concentration of defects in Cu_2_O thin films. In our case, the long lifetime obtained for films deposited with no oxygen implies the presence of larger complex defects than previously reported for Cu_2_O. As the oxygen fraction increases, the overall concentration of defects decreases, in particular *V*_O_, thus explaining the evolution of the transport properties. Our results represent a fundamental step towards reducing the gap in performance between Cu_2_O thin films deposited by physical methods and chemical methods, thus opening the door to the chemical fabrication of efficient Cu_2_O-based devices.

## Methods

### Deposition of Cu_2_O thin films

Cuprous oxides were deposited using a home-made spatial atomic layer deposition reactor^38^. (Cu(hfac)(cod)) and water vapor were used as a metal precursor and oxidizing agent. The precursor bubbler and the gas transport pipe were maintained at 90 °C and 95 °C, respectively. The metal precursor was delivered with a mass flow of 60 sccm, and diluted in an additional 60 sccm flow of nitrogen. Four additional lines were used to separate the metal Cu precursor from the oxidant flow, with a total nitrogen flow of 648 sccm. The nitrogen line is crucial since pure oxygen was added to the Cu_2_O thin films with several fractions (5–50%). Water was carried using a mass flow of 120 sccm, transported in an extra N_2_ flow of 120 sccm from two lines, each delivering 120 sccm to the glass substrate. The borosilicate glass substrates (surface area of 3×5 cm²) were cleaned with isopropanol for 5 min in an ultrasonic bath and then dried with N_2_. The temperature deposition was then varied from 180 °C to 260 °C with an incremental temperature value of 20 °C between the different samples.

### Analysis techniques

X-ray diffraction data was achieved with a Bruker D8 Advance diffractometer in the Bragg-Brentano (θ–2θ) configuration, with Cu K_α1_ radiation (0.15406 nm), to define the crystallinity of the layers. Grazing Incidence X-ray Diffraction (GI-XRD-Coplanar grazing incidence X-ray diffraction) acquisitions were collected on a RIGAKU Smartlab equipped with a 9 kW rotating anode Cu source (45 kV and 200 mA). From the XRD patterns, the crystallite size and FWHM if the Cu_2_O thin films were calculated from the Debye-Scherer equation^66^:

1 $$\tau=\frac{K\lambda }{\beta \,\cos \theta }$$

Where *θ* is the experimental Bragg angle of the Cu_2_O diffraction peak, *β* is the full width at half-maximum (FWHM), K is the shape factor equal to 0.9. Atomic Force Microscopy (AFM) was performed in tapping mode using a Digital instrument D3100 Nanoscope. A Jobin Yvon/Horiba LabRam spectrometer was used to realize Raman measurements covering the range from 50 to 700 cm^−1^ with a blue laser having a wavelength of 488 nm. X-Ray Photoelectron spectra were recorded on a ThermoScientific K-Alpha spectrometer using a monochromatized Al Kα radiation source (1486.6 eV), in ultra-high vacuum (10^−8^ mbar) at room temperature. The X-Ray beam area (spot size) was adjusted to 400 μm in diameter. An in situ Ar ion gun (2 keV) was used for 60 s in order to remove the surface contamination of the films. The spectra were acquired in the constant analyzer energy mode using a pass energy of 30 eV and a step size of 0.1 eV for these core levels. A 100 eV pass energy has been used for the general survey spectrum (step 0.5 eV). The spectra shown in the figures have been averaged over 3 scans for the survey and 10 scans for the core levels. The optical properties were investigated with a lambda 950 spectrophotometer from Perkin Elmer. The optical bandgap of the different films was extracted from the transmittance and reflectance spectra by fitting of the Tauc plots in which (αhυ)^2^ is plotted versus (hυ), where hυ is the photon energy and a is the absorbance calculated from the transmittance data (*α* = (log(10)/L)*(log(100 − *R*) − log(T))), where *L*, *R*, and *T* represents the thickness, reflectance, and transmittance of the film respectively. The surface morphology and thickness of the films were characterized using a scanning electron microscopy (SEM-FEG) GeminiSEM 300 instrument and confirmed by Ellipsometer. Film thickness was also measured by X-ray reflectometry (XRR, D500 Siemens model, using Cu Kα radiation (*λ* = 0.15406 nm), 0.01°/step, 2 s/step). The electrical properties were measured with a Keithley 2400 source meter connected to a 4-point probe station in the Van der Pauw configuration at room temperature. A Hall Effect setup with a magnetic field of 0.5 Tesla was used to measure the transport properties on 1 × 1 cm² samples with evaporated gold contact on the top. Transmission electron microscopy (TEM) observations were performed at 200 kV with a JEOL 2010 microscope (with a resolution of ~0.19 nm). Cross-sectional samples were prepared by automated polishing, the latter using the MultiPrepTM system (Allied High Tech Products, Inc.). The final polishing was performed using a felt-covered disc impregnated with a silica solution until the appearance of the first extinction fringe among those of equal thickness. Ar-ion milling was then used to minimize the total thickness. Electron diffraction patterns were obtained in plane view by scratching the surface of the Cu_2_O with a diamond tip. The residues were put on a carbon film placed over a copper grid to prepare the specimens for transmission electron microscopy (TEM). Doppler broadening variable energy positron annihilation spectroscopy (DB-VEPAS) measurements were conducted at the apparatus for in situ defect analysis (AIDA)^67^ of the slow positron beamline (SPONSOR)^68^. The positron annihilation lifetime spectroscopy (PALS) experiments were performed at the mono-energetic positron spectroscopy (MePS) beamline, which is the end station of the radiation source ELBE (Electron Linac for beams with high Brilliance and low Emittance) at HZDR (Germany)^39^. A CeBr_3_ scintillator detector and a SPDevices ADQ14DC-2X digitizer (14-bit / 2GS/s) were used for digital data acquisition and annihilation events analysis^69^. Positron lifetimes for the delocalized (bulk lifetime) and localized states—positrons trapped at vacancy-like defects and their agglomerations were calculated using the atomic superposition (ATSUP) method within two-component density functional theory (DFT) ab initio calculations^70^. For the electron-positron correlation, the generalized gradient approximation (GGA) scheme with a gradient correction (GC) was used^71^. More details about positron techniques and DFT calculations are provided in Supplementary note 2.2.

### Reporting summary

Further information on research design is available in the Nature Research Reporting Summary linked to this article.

## Supplementary information

Supplementary Information

Lasing Reporting Summary

Reporting Summary

## Data Availability

The experimental raw data that support the findings of this study are available from the corresponding author upon reasonable request.
