# Supplementary material for: Chemical deposition of Cu2O films with ultra-low resistivity: correlation with the defect landscape
Source: Nat Commun. 2022 Sep 9;13:5322. doi: 10.1038/s41467-022-32943-4 (PMC9463139; doi:10.1038/s41467-022-32943-4)
Supplement: Supplementary file 1 — Supplementary Information [file 41467_2022_32943_MOESM1_ESM.pdf]

Supplementary Information

Chemical deposition of Cu_2O films with ultra-low resistivity: Correlation with the defect landscape

Abderrahime Sekkat[1,2,3], Maciej Oskar Liedke[4], Viet Huong Nguyen[5], Maik Butterling[4], Federico Baiutti[6], Juan de Dios Sirvent Veru[6], Matthieu Weber[1], Laetitia Rapenne[1], Daniel Bellet[1], Guy Chichignoud[3], Anne Kaminski-Cachopo[2], Eric Hirschmann[4], Andreas Wagner[4], and David Muñoz-Rojas[1,*]*

[1] Univ. Grenoble Alpes, CNRS, Grenoble INP, LMGP, F-38000 Grenoble, France
[2] Univ. Grenoble Alpes, Univ. Savoie Mont Blanc, CNRS, Grenoble INP, IMEP-LaHC, 38000 Grenoble, France
[3] Univ. Grenoble Alpes, CNRS, Grenoble INP, SIMAP, 38000 Grenoble, France.
[4] Institute of Radiation Physics, Helmholtz-Zentrum Dresden-Rossendorf, Bautzner Landstrasse 400, 01328, Dresden, Germany
[5] Faculty of Materials Science and Engineering, Phenikaa University, Hanoi 12116, Vietnam
[6] Catalonia Institute for Energy Research (IREC), Jardins de Les Dones de Negre 1, Barcelona 08930, Spain

Corresponding authors:
david.munoz-rojas@grenoble-inp.fr ; abderrahime.sekkat@grenoble-inp.fr

1. Effect of Deposition Temperature

Deposition temperature was varied from 180 °C to 260 °C by an incremental value of 20 °C between each sample at an oxygen fraction of 15% related to the lowest resistivity value obtained, which is 2 Ω.cm. **Fig.S1a** shows the variation of the resistivity value from 483 Ω.cm at 180 °C to 0.9 Ω.cm at 260 °C. This variation is allocated to a significant reduction in the energy formation of defects in an oxygen-rich atmosphere due to the increase of the thermal budget during the deposition process.

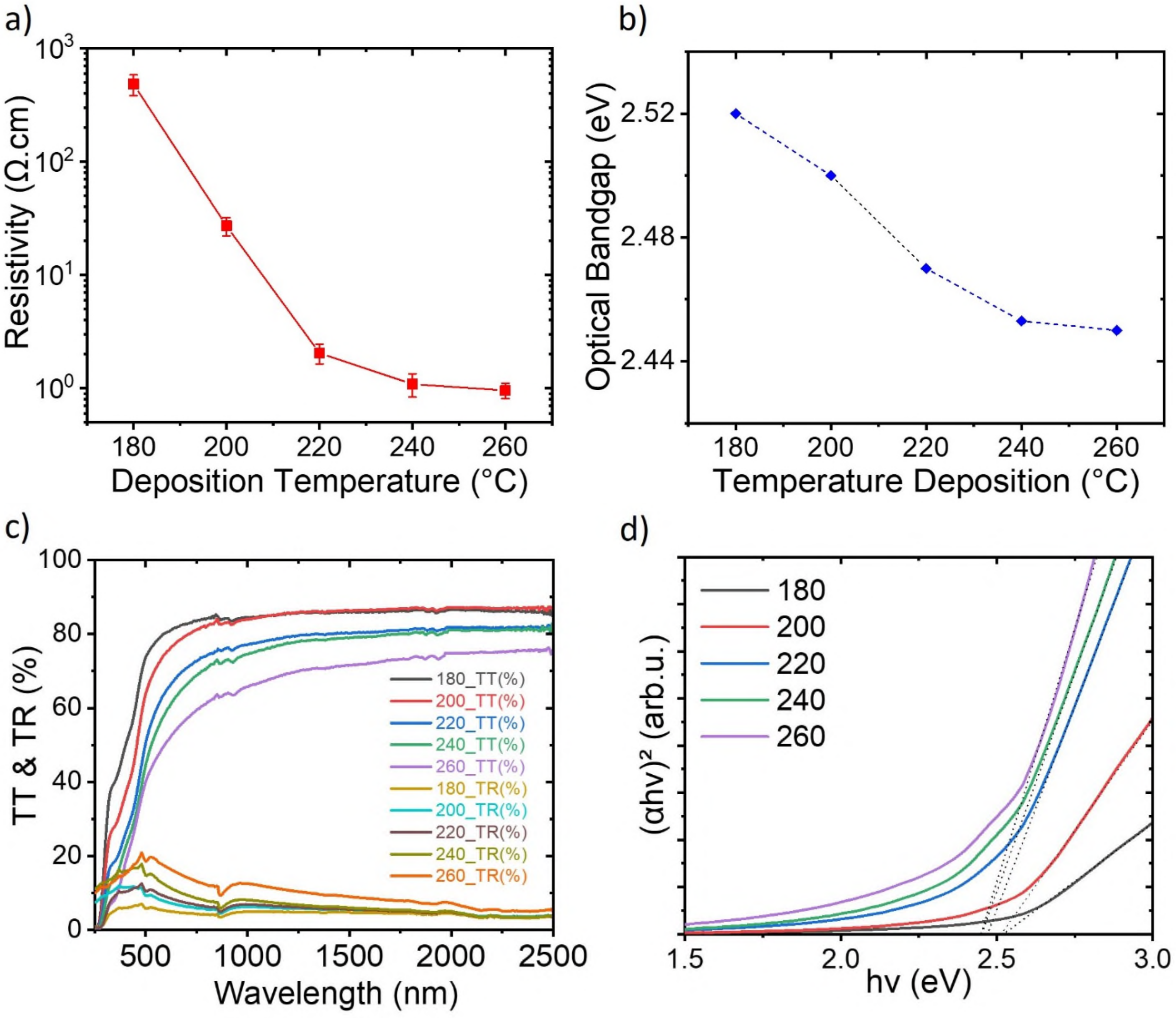

***Supplementary Fig.1**. a). Van der Pauw measurements, b). Optical bandgap, c). Total transmittance, and reflectance, and d). Tauc plot measurement of Cu_2O, deposited on Glass substrate at 15% oxygen fraction at different deposition temperatures (180 °C – 260 °C).*

The optical direct allowed band gap was then extracted from total transmittance and reflectance measurements of those samples using a Tauc plot fitting as shown in **FigS1.b-d**. A decrease of the bandgap from 2.52 eV to 2.44 eV is observed and it is mainly related to the extension of the absorption in the UV region with the increase of deposition temperature. This may imply that the films promote a change in the defect formation caused by the variation of deposition temperature as shown in similar results that were reported in our previous work.[1] In fact, the deposition temperature will also promote, in an oxygen-rich atmosphere, the high formation energy of most defects, namely copper vacancies in the normal and split configuration. Indeed, recent work has shown that the impact of the crystallinity and grain boundaries are not the main aspect affecting the transport properties of the film as compared to the impact of defect formation.[2]

2. Thin Film Characterization

2.1. Optical properties, XPS, and XRR analyses for the Cu_2O thin film deposited at 260 °C with and oxygen fraction of 15%.

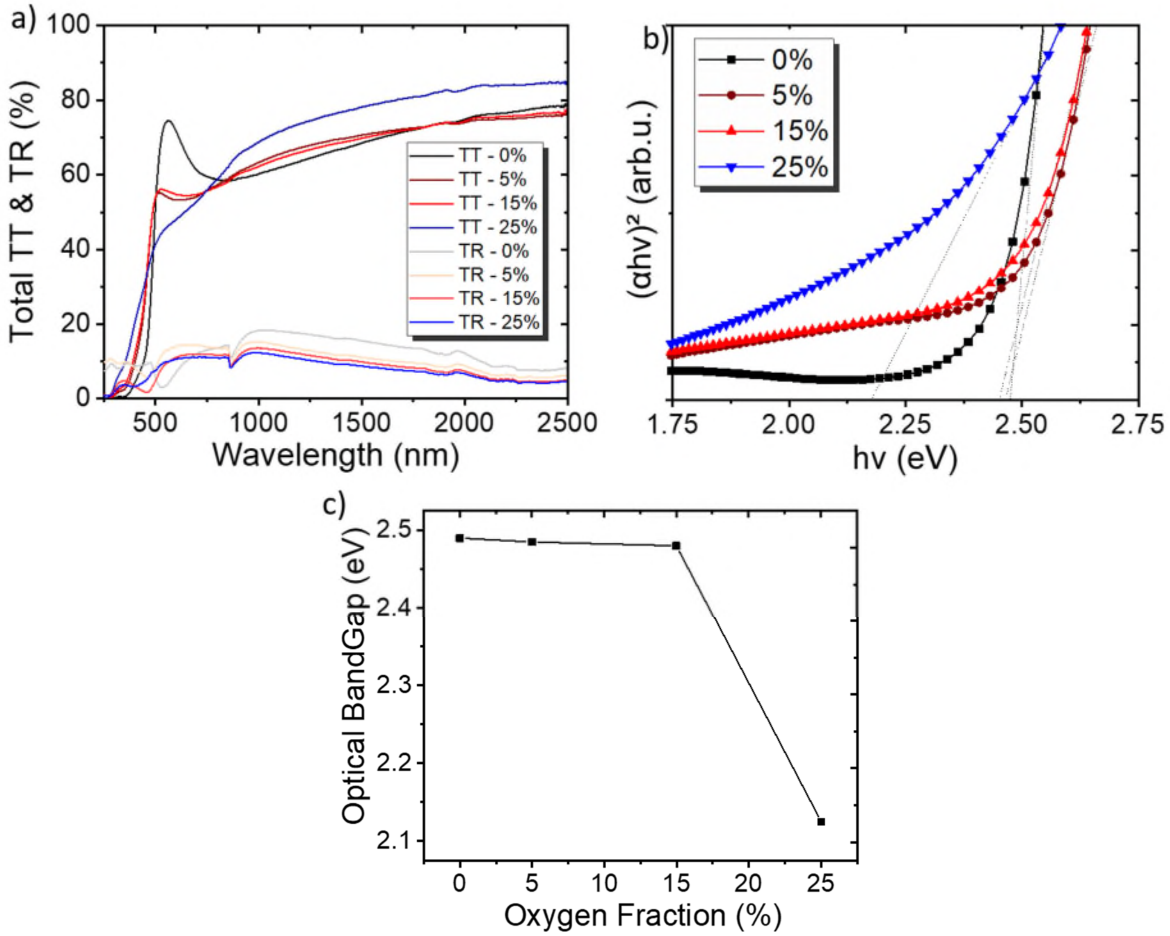

Supplementary Fig.2. *a). Total transmittance, and reflectance, b). Tauc plot measurement of Cu_2O, and c) extracted optical bandgap, deposited on Glass substrate at 260 °C with different oxygen fraction.*

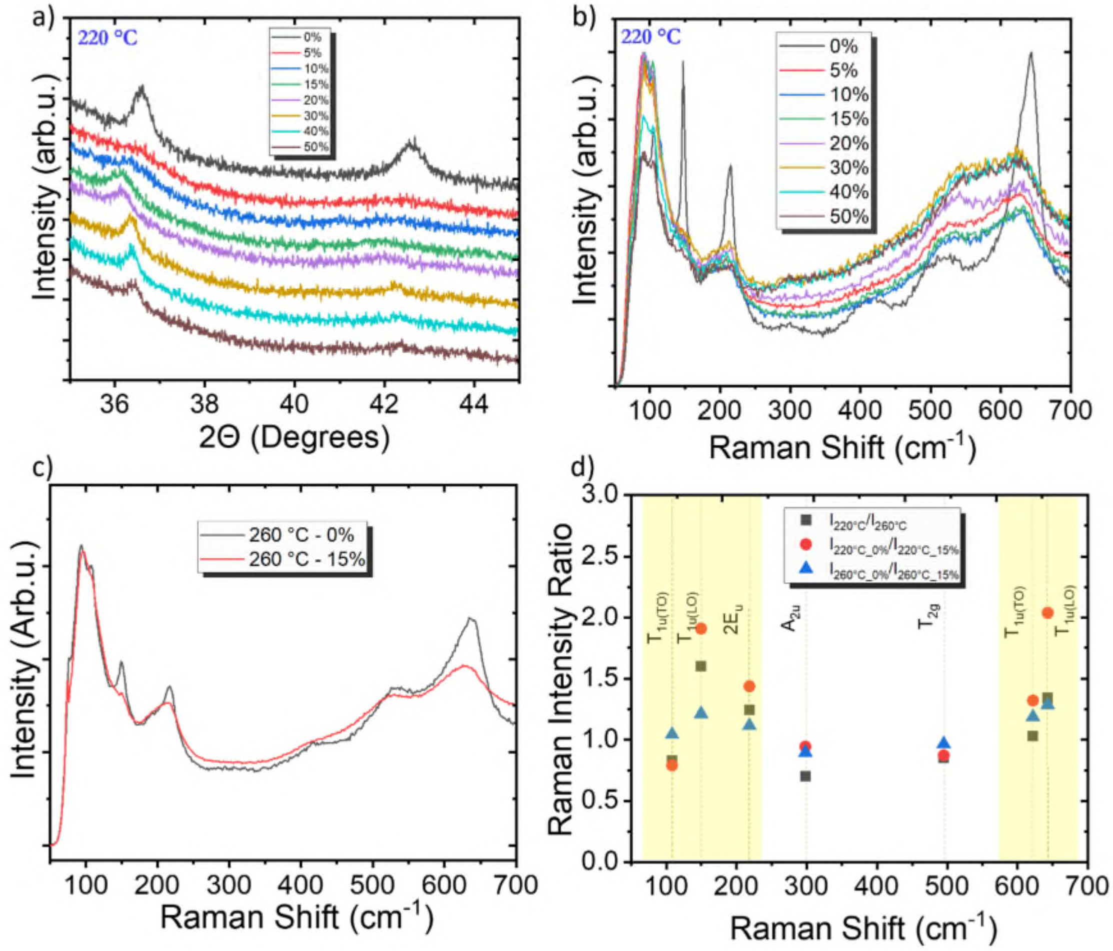

***Supplementary Fig.3**. a). XRD pattern, and Raman Measurement, of Cu_2O thin films deposited b). at 220 °C with different oxygen fractions, c). at 260 °C with 0% and 15% oxygen fraction on a borosilicate glass, d). Intensity Ratio of several Raman modes between samples deposited at 220 °C with 0% and 15% oxygen ratio, at 260 °C with 0% and 15% oxygen ratio, and at 220 °C and 260 °C both at 0% oxygen ratio*

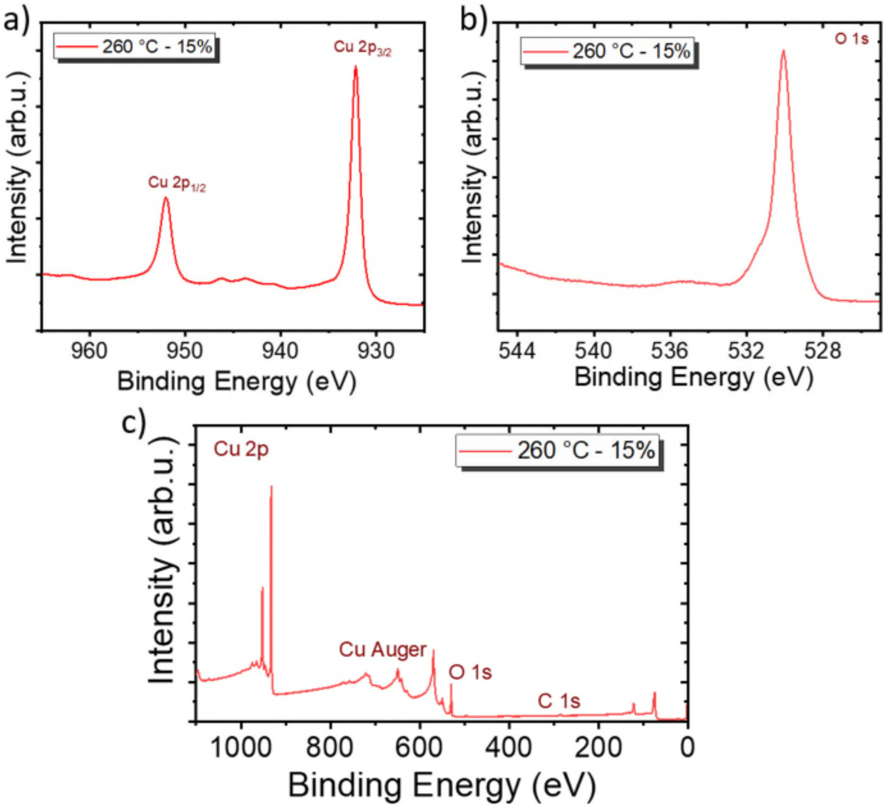

Supplementary ***Fig.4***. *XPS spectra, of a). Cu 2p, b). O 1s, and c). survey of* Cu_2O *deposited at 260 °C with 15% oxygen fraction on Glass substrate.*

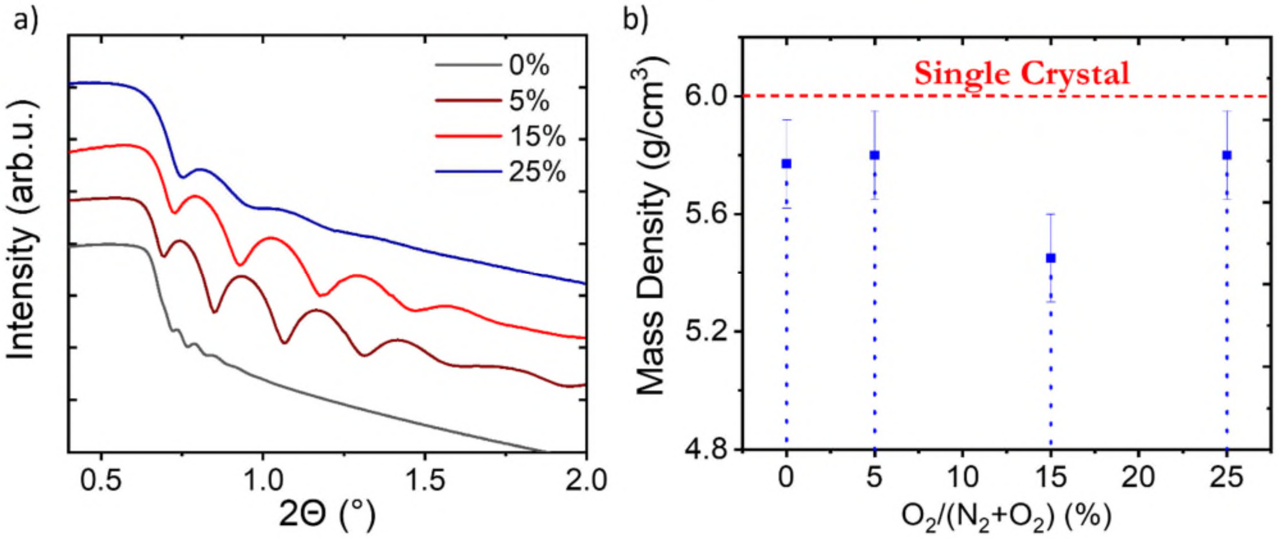

Supplementary Fig.5. a). XRR patterns, and, b). Extracted mass density (g/cm^3), of Cu_2O deposited at 260 °C with a different oxygen fraction. The variation of critical angle indicates the change in the mass density of the deposited films.

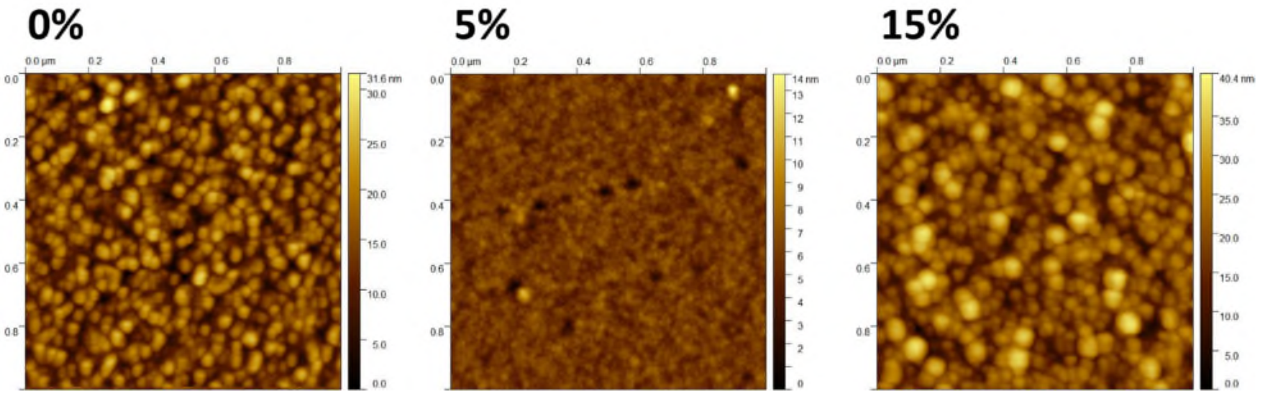

Supplementary Fig.6. AFM topography images of Cu_2O thin films deposited on glass at 260 °C with different oxygen fraction (0%, 5%, and 15%)

Supplementary Table .1. FWHM of samples deposited at 260 °C with 0%, 5%, and 15% oxygen fraction

**Sample**		**FWHM**	**Crystallite Size (nm)**
**260 °C**	**0%**	1.05106	7.95
	**5%**	1.0083	8.29
	**15%**	0.70868	11.80

2.2. Positron Annihilation Spectroscopy (PAS)

2.2.1. Doppler Broadening Variable Energy Positron Annihilation Spectroscopy (DB-VEPAS)

Doppler broadening variable energy positron annihilation spectroscopy (DB-VEPAS) measurements have been conducted at the apparatus for in-situ defect analysis (AIDA)[3] of the slow positron beamline (SPONSOR).[4] Positrons have been accelerated to discrete kinetic energies E_p in the range between 0.05 and 35keV and implanted into samples at depths as shallow as the surface down to couple of micrometers. A mean positron implantation depth can be approximated by a simple material density dependent formula: $\langle z\rangle=36/\rho\cdot E_p^{1.62}$, where $\rho=6.01\ g\cdot cm^{-3}$ is the density of Cu_2O.[5] During the implantation process positrons lose their kinetic energy due to thermalization and after short diffusion annihilate in delocalized lattice sites or are trapped in vacancy like defects and their agglomerations as well as in dislocations. Once they encounter electrons emission usually

two anti-collinear 511keV gamma photons follows the annihilation. Since at the annihilation site thermalized positrons have very small momentum compare to the electrons a broadening of the 511 keV line is observed mostly due to momentum of the electrons.

2.2.2. Calculation of the positron diffusion length using VEPFit

For the analysis of positron diffusion length, L_+, which is inverse proportional to defect concentration the VEPFit code[6] was utilized. It permits to fit $S(E_p)$ curves for multilayered systems and to acquire thickness, L_+, and specific S-parameters for each layer within a stack. The calculated defected layer thickness, S- parameters, and L_+ are presented in **Table 1**. The following material densities have been utilized for Cu_2O and BS-glass: ρ_{Cu_2O}=5.45-5.8 g·cm^{-3} (given by the XRD analysis) and $\rho_{BS\text{-}glass}$=2.23 g·cm^{-3}, respectively. L_+ for the substrate was acquired from the measurement of the back side of the 260°C – 15% O_2 sample and fixed to 41.72 nm for all the other samples.

The 0% sample has largest S and lowest L_+ hence large defect concentration. S is decreasing and L_+ increasing with the oxygen fraction suggesting a strong (8-10 times) decrease of defect concentration. The calculated defected layer thickness is close to the nominal besides for the 5% sample, where it was impossible to fit the curve with a larger thickness. Existence of another defected layer closer to the interface with the substrate would explain the discrepancy.

2.2.3. Positron Annihilation Lifetime Spectroscopy (PALS)

The positron annihilation lifetime experiments were performed at the mono-energetic positron spectroscopy (MePS) beamline, which is the end station of the radiation source ELBE (Electron Linac for beams with high Brilliance and low Emittance) at HZDR (Germany)[7] using a digital lifetime $CrBr_3$ scintillator detector with a dedicated software employing a SPDevices ADQ14DC-2X digitizer with 14 bit vertical resolution and 2GS/s horizontal resolution[8] and with a time resolution function down to about 0.230 ns. The resolution function required for spectrum analysis uses typically two Gaussian functions (the so-called resolution functions) with distinct E_p dependent intensities and relative shifts. Typical lifetime spectrum N(t) is described by $N(t)=\Sigma (1/\tau_i) I_i \exp(-t/\tau_i)$, where τ_i and I_i are the positron lifetime and intensity of the i-th component, respectively ($\Sigma I_i=1$). All spectra contained at least $1\cdot10^7$ counts. The spectra were deconvoluted using the non-linearly least-squared based package PALSfit fitting software[9] into 3 discrete lifetime components (τ_1-τ_3), which directly evidence 3 different defect types (sizes). The corresponding relative intensities (I_1-I_3) reflect relative changes of each defect type (size) density. The second and the third lifetime component originate from larger vacancy clusters at grain boundaries and surface states, respectively. The relative intensity I_3 of the third component is below 0.5-1%, hence negligibly small. In general, positron lifetime is directly proportional to defects size, i.e., the larger is the open volume, the lower is the probability and longer it takes for positrons to be annihilated

with electrons.[10,11] The positron lifetime and its intensity has been probed in function of positron implantation energy E_p or in the other words implantation depth (thickness).

2.2.4. ATomic SUPerposition DFT calculations (ATSUP)

Theoretical calculations of positron lifetimes for the delocalized (bulk lifetime) and localized states trapped at vacancy like defects and their agglomerations were obtained using the atomic superposition (ATSUP) method within two-component density functional theory (DFT) *ab initio* calculations.[12] For the electron-positron correlation, the generalized gradient approximation (GGA) scheme with a gradient correction (GC) was used.[13] The gradient correction scheme is more sensitive to details of the electronic structure compare to the other schemes, e.g. to Boronski-Nieminen (BN) approach. For calculations, the cuprite lattice structure of Cu_2O (space group 224, Pn-3m) with six atoms in the unit cell and a lattice constant of 0.4267 nm was used. The supercell is constructed of 4 × 4 × 4 unit cells (384 atoms in total). Different vacancy configurations were simulated including Cu (V_{Cu}), oxygen (V_O), and split vacancies ($V_{Cu,split}$), as well as their complexes. The calculated positron lifetimes corresponding to this states are presented in **Table S2**.

2.2.5. Calculation of defect concentration based on positron diffusion length

Assuming that the films mostly contain one major defects type (as indicated by the PALS analysis) the vacancy concentration C_V can by calculated as follows:[14]

$$\boldsymbol{c_V = \frac{1}{\nu_V \tau_B}\left(\frac{L_{+,B}^2}{L_+^2} - 1\right)},$$

where ν_V is a specific positron trapping rate (trapping coefficient), τ_B and $L_{+,B}$ are a bulk lifetime and a diffusion length in a defects free material. The latter can be calculated as

$L_{+,B} = \sqrt{D_+ \tau_B}$, where D_+ is the room temperature positron diffusion coefficient.

According to our ATSUP calculations (see section 2.2.5) $\tau_B \approx 180$ ns was utilized, which possibly is slightly overestimated.[15] The values of D_+ are often not available in literature. For semiconductors diffusion coefficient is typically in the range $D_+ = 1\text{-}2\ cm^2s^{-1}$.[11] The known values for metals, for example for Al and Cu $D_{+,Al} = 1.7\pm0.2\ cm^2s^{-1}$ and $D_{+,Cu} = 1.7\pm0.5\ cm^2s^{-1}$ are found in the similar range.[16] Utilizing D_+ value of Cu $L_{+,B}$=174.9 nm is obtained. On the other hand, specific positron trapping rate in semiconductors is $\nu_V \approx 2\cdot10^{15}\ s^{-1}$ for single vacancies[17] and $N\cdot\nu_V$ for N number of vacancies within a complex[18] or the N number of neutral pairs of metal vacancy – oxide vacancy complexes.[19] In our case at least N=2 is expected, hence $\nu_V \approx 4\cdot10^{15}\ s^{-1}$ was taken to calculate defect concentration. However, assuming that V_O is not neutral within a complex N=3-4 should be taken. It is suggested by our ATSUP calculations by increased positron lifetime once V_O is added to V_{Cu}. The results of the ATSUP calculations are presented in **Table S2**. As shown, each defect has associated a particular positron lifetime and a binding energy. The lifetime is related to the volume of the calculated defect while the binding energy provides an indication of the affinity

of the positrons for a particular defect (i.e. the lower the binding energy, the lower the affinity and thus the lower the probability to annihilate in that particular defect).

Supplementary Table.2. *Calculated using ATSUP code positron lifetimes for different vacancy configurations in Cu_2O crystal.*

annihilation site	positron lifetime (ps)	Positron binding energy (eV)
bulk	180.22	-
V_{Cu}	229.24	0.327
$V_{Cu,split}$	190.54	0.099
V_O	180.83	0.013
V_{Cu} - V_O	262.22	0.678
$V_{Cu,split}$ - V_O	210.58	0.245
V_{2Cu}	256.34	0.722
V_{2Cu} - V_O	271.92	0.916
V_{Cu} - $V_{Cu,split}$ - V_O	240.09	0.584
V_{2Cu} - $V_{Cu,split}$ - V_O	269.78	0.966
V_{3Cu}	274.43	0.978
V_{4Cu}	296.10	1.216
V_{2Cu} – V_{2O}	313.52	1.356

2.3. Formed Defects in Cu_2O Thin Films

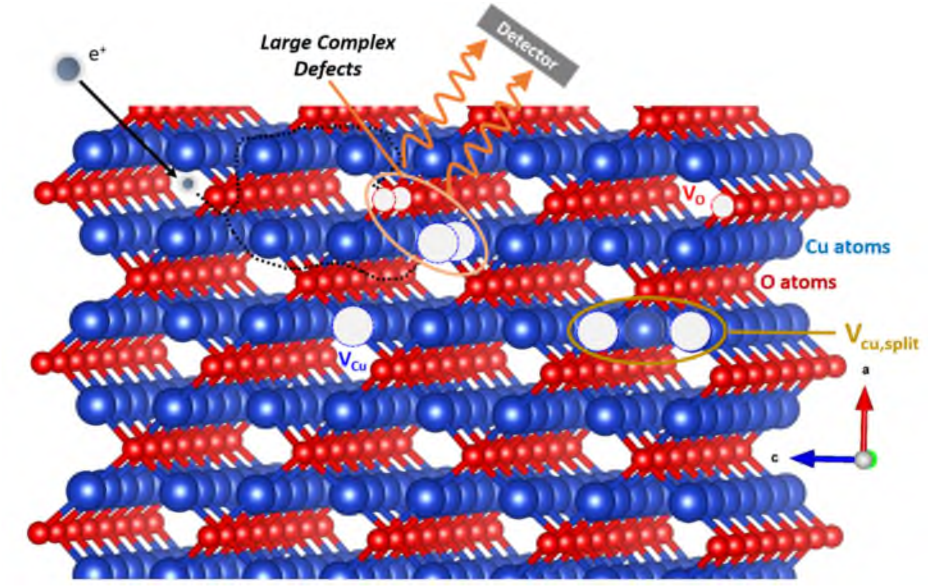

Supplementary Fig.7. Schematic model of the different point defects probed with the positron in the PALS, including a copper vacancy in the normal and split configuration (V_{Cu}, $V_{Cu,split}$), oxygen vacancy (V_O), and complex defects (V_{2Cu}-V_{2O}) (Blue and red atoms represent the Cu and O atoms, *respectively - Vesta)*

Supplementary References

1. Sekkat, A. *et al.* Open-air printing of Cu_2O thin films with high hole mobility for semitransparent solar harvesters. *Commun. Mater.* **2**, (2021).
2. Aggarwal, G., Maurya, S. K., Singh, A. J., Singh, A. K. & Kavaipatti, B. Intrinsic acceptor-like defects and their effect on carrier transport in polycrystalline Cu_2O photocathodes. *J. Phys. Chem. C* **123**, 26057–26064 (2019).
3. Liedke, M. O. *et al.* Open volume defects and magnetic phase transition in $Fe_{60}Al_{40}$ transition metal aluminide. *J. Appl. Phys.* **117**, (2015).
4. Anwanda, W., Brauer, G., Butterling, M., Kissener, H. R. & Wagner, A. Design and construction of a slow positron beam for solid and surface investigations. *Defect Diffus. Forum* **331**, 25–40 (2012).
5. Dryzek, J. & Horodek, P. GEANT4 simulation of slow positron beam implantation profiles. *Nucl. Instruments Methods Phys. Res. Sect. B Beam Interact. with Mater. Atoms* **266**, 4000–4009 (2008).
6. van Veen, A. *et al.* VEPFIT applied to depth profiling problems. *Appl. Surf. Sci.* **85**, 216–224 (1995).
7. Wagner, A., Butterling, M., Liedke, M. O., Potzger, K. & Krause-Rehberg, R. Positron annihilation lifetime and Doppler broadening spectroscopy at the ELBE facility. *AIP Conf. Proc.* **1970**, (2018).
8. Hirschmann, E. *et al.* A new system for real-time data acquisition and pulse parameterization for digital positron annihilation lifetime spectrometers with high repetition rates. *J. Instrum.* **16**, P08001 (2021).
9. Olsen, J. V., Kirkegaard, P., Pedersen, N. J. & Eldrup, M. PALSfit: A new program for the evaluation of positron lifetime spectra. *Phys. status solidi* **4**, 4004–4006 (2007).
10. Krause-Rehberg, R. & Leipner, H. S. *Positron annihilation in semiconductors: defect studies*. (1999).
11. Tuomisto, F. & Makkonen, I. Defect identification in semiconductors with positron annihilation: Experiment and theory. *Rev. Mod. Phys.* **85**, 1583–1631 (2013).
12. Puska, M. J. & Nieminen, R. M. Theory of positrons in solids and on solid surfaces. *Rev. Mod. Phys.* **66**, 841–897 (1994).
13. Alatalo, M. *et al.* Theoretical and experimental study of positron annihilation with core electrons in solids. *Phys. Rev. B* **54**, 2397–2409 (1996).

14. Sherrer, P. Bestimmung der Grösse und der Inneren Struktur von Kolloidteilchen Mittels Röntgenstrahlen, Nachrichten von der Gesellschaft der Wissenschaften, Göttingen. *Math. Klasse* **2**, 98–100 (1918).

15. Kuriplach, J. & Barbiellini, B. Gradient correction scheme for bulk and defect positron states in materials: New developments. *J. Phys. Conf. Ser.* **505**, 012040 (2014).

16. Soininen, E. *et al.* Temperature dependence of positron diffusion in cubic metals. *Phys. Rev. B* **41**, 6227–6233 (1990).

17. Saarinen, K., Hautojärvi, P. & Corbel, C. Chapter 5 Positron Annihilation Spectroscopy of Defects in Semiconductors. in *Semiconductors and Semimetals* vol. 51 209–285 (Elsevier, 1998).

18. Nieminen, R. M. & Laakkonen, J. Positron trapping rate into vacancy clusters. *Appl. Phys.* **20**, 181–184 (1979).

19. Zubiaga, A. *et al.* Positron annihilation lifetime spectroscopy of ZnO bulk samples. *Phys. Rev. B* **76**, 085202 (2007).
